# Evaluating the Capacity to Generate and Preserve Nitric Oxide Bioactivity in Highly Purified Earthworm Erythrocruorin: A GIANT POLYMERIC HEMOGLOBIN WITH POTENTIAL BLOOD SUBSTITUTE PROPERTIES*

**DOI:** 10.1074/jbc.M114.583260

**Published:** 2014-11-04

**Authors:** Camille J. Roche, Abhinav Talwar, Andre F. Palmer, Pedro Cabrales, Gary Gerfen, Joel M. Friedman

**Affiliations:** From the ‡ Department of Physiology and Biophysics, Albert Einstein College of Medicine, Bronx, New York 10461,; § Herricks High School, New Hyde Park, New York 11040,; ¶ William G. Lawrie Department of Chemical and Biomolecular Engineering, The Ohio State University, Columbus, Ohio 43210, and; ‖ Department of Bioengineering, University of California, San Diego, La Jolla, California 92093-0412

**Keywords:** Hemoglobin, Myoglobin, Nitric Oxide, Oxidation-Reduction (Redox), Reductase, Dinitrogen Trioxide, Erythrocruorin, Nitrite Anhydrase, Nitrite Reductase

## Abstract

The giant extracellular hemoglobin (erythrocruorin) from the earth worm (*Lumbricus terrestris*) has shown promise as a potential hemoglobin-based oxygen carrier (HBOC) in *in vivo* animal studies. An important beneficial characteristic of this hemoglobin (LtHb) is the large number of heme-based oxygen transport sites that helps overcome issues of osmotic stress when attempting to provide enough material for efficient oxygen delivery. A potentially important additional property is the capacity of the HBOC either to generate nitric oxide (NO) or to preserve NO bioactivity to compensate for decreased levels of NO in the circulation. The present study compares the NO-generating and NO bioactivity-preserving capability of LtHb with that of human adult hemoglobin (HbA) through several reactions including the nitrite reductase, reductive nitrosylation, and still controversial nitrite anhydrase reactions. An assignment of a heme-bound dinitrogen trioxide as the stable intermediate associated with the nitrite anhydrase reaction in both LtHb and HbA is supported based on functional and EPR spectroscopic studies. The role of the redox potential as a factor contributing to the NO-generating activity of these two proteins is evaluated. The results show that LtHb undergoes the same reactions as HbA and that the reduced efficacy for these reactions for LtHb relative to HbA is consistent with the much higher redox potential of LtHb. Evidence of functional heterogeneity in LtHb is explained in terms of the large difference in the redox potential of the isolated subunits.

## Teaser

**Background:** Earthworm hemoglobin (LtHb) is a potential blood substitute.

**Results:** LtHb can generate nitric oxide (NO) and preserve NO bioactivity.

**Conclusion:** LtHb reactions with nitrite and NO are indicative of therapeutic possibilities.

**Significance:** The results further highlight the potential role of hemoglobins in NO homeostasis.

## Introduction

Acellular hemoglobins as hemoglobin-based oxygen carriers (HBOCs)^2^ have been the focus of attempts to develop oxygen transport therapeutics that could effectively replace conventional red blood cell-based transfusions. However, no Food and Drug Administration-approved product exists because of the inherent limitations associated with infusion of acellular hemoglobins as HBOCs. One limiting factor is the low number of oxygen binding sites in most HBOCs such as human adult hemoglobin (HbA) that restricts the amount of protein that can be infused into the body because of the potential for high vascular osmotic stress. The second limiting factor is HBOC toxicity. A major source of this toxicity comes from efficient nitric oxide (NO) scavenging by acellular Hbs (1, 2). The NO scavenging occurs primarily through the nitric oxide dioxygenase reaction in which oxygen bound to the heme reacts with circulating NO to form nitrate and ferric heme. NO depletion through this reaction directly causes proinflammatory conditions including vasoconstriction and enhanced platelet aggregation, defeating the purpose of the transfusion. The last form of toxicity of HBOCs comes from met heme, which can form from either the nitric oxide dioxygenase reaction or the autoxidation of oxygen-bound heme that also generates reactive oxygen species (3, 4). Both met heme and reactive oxygen species can be proinflammatory.

Thus, to be effective, an HBOC requires properties such that it counteracts high osmotic stress, low oxygen carrying capacity, and NO scavenging as well as any other acellular Hb-derived toxicity. Attempts to overcome the osmotic issue have taken the form of generating polymeric forms of mammalian hemoglobins; however, most still scavenge NO and are vasoactive. The NO scavenging problem can in principle be overcome through recombinant technology that generates Hbs with low rates of nitric oxide dioxygenase (1). Alternatively it can be addressed through several proposed and studied hemoglobin-based reactions with nitrite (5–11) that can compensate for NO scavenging by generating NO and related bioactive forms of NO molecules or stable Hb species that preserve NO bioactivity (12–14).

In the quest for suitable blood substitutes, one candidate that has drawn some attention is the hemoglobin from the earthworm (*Lumbricus terrestris*) (15–25). A closely related worm hemoglobin from *Arenicola marina* is currently being evaluated as an HBOC in France (16, 26). The acellular hemoglobins from these worms are very large polymeric hemoglobins with many oxygen binding sites. The acellular hemoglobin from the earth worm (LtHb) is of interest because each molecule contains 144 oxygen binding hemes, significantly more than HbA, which contains only four. In addition, LtHb has a high redox potential (27). The redox potential is a measure of the tendency of the heme to undergo reduction. The high redox potential is a likely origin of the high stability of LtHb as a ferrous species (low rate of oxidation/met formation). Unlike HbA, LtHb does not readily dissociate into smaller polymeric units upon dilution under physiological conditions. These factors are all positive with respect to efficacy as a potent HBOC. Most significant is that the large oxygen carrying capacity of each molecule permits the use of much lower concentrations of infused protein to achieve needed therapeutic levels of delivered oxygen. As a result, the use of LtHb overcomes the osmotic limitations that prevent achieving therapeutic levels of oxygen when infusing HBOCs with much fewer oxygen binding sites. *In vivo* studies (17, 18) have demonstrated that LtHb can be effective in delivering oxygen without obvious toxic consequences. Additionally there is evidence (18, 26) that the rate for the nitric oxide dioxygenase reaction for both of these multimeric giant Hbs is much lower than for HbA, suggesting that NO scavenging many not be as big an issue for these species if used as HBOCs. The current project addresses the remaining important issue of the ability of LtHb to overcome any potential NO scavenging through compensatory reactions that generate NO and long lived forms of NO. There is growing evidence that acellular Hbs from many invertebrates play a role in NO-related homeostasis (28, 29).

One reaction often invoked as a possible mechanism of generating NO from Hbs is the nitrite reductase (NR) reaction (8, 10, 11) (Reaction 1), which results in the production of NO from the reaction of five-coordinate ferrous hemoglobin (*i.e.* deoxyhemoglobin) and nitrite.

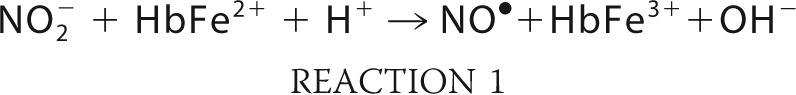
 The rate of this reaction for tetrameric hemoglobin is dependent upon the quaternary state of the protein (30–32). It has been shown that R state proteins undergo the NR reaction much faster than T state proteins. The dependence of this reaction on quaternary state is attributable at least in part to the effect of quaternary state on the redox potential of the heme (31, 33, 34). A high redox potential indicates that the reduced (Fe^2+^) form of heme is favored. The T state structure of HbA has a higher redox potential relative to the R state. As a consequence, the NR reaction, which involves going from the reduced form of heme to the oxidized form, is favored by the lower redox potential of the R state. The implication is that heme proteins with higher redox potentials will have lower NR rates. Furthermore, the NR reaction appears to be particularly important for HBOC studies: HBOCs that display high NR rates generally show the lowest levels of induced vasoconstriction (35). However, for NO-related reactions to counterbalance scavenging, it is likely that the Hb must generate either long lasting bioactive forms of NO as opposed to the very short lived vascular NO or form derivatives that preserve NO bioactivity. In this respect, the NR reaction appears inadequate and is likely to be only one part of a larger process. Most recently it has been shown that this reaction, occurring in red blood cells, is associated with processes that reduce platelet activity (decreases activation), consistent with either free NO or an *S*-nitrosothiol-containing species being the active agent (36, 37).

A proposed reaction responsible for the generation of long lived forms of bioactive NO is the nitrite anhydrase (NA) reaction (7, 8, 10, 38) (Reactions 2 and 3).

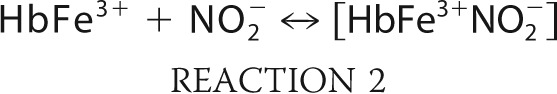


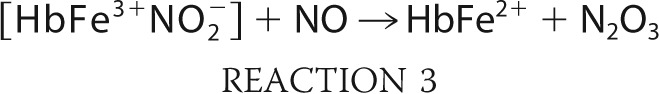
 The significance of this reaction is that N_2_O_3_ is a potent *S*-nitrosating agent of thiol-containing molecules such as glutathione (GSH). The resulting *S*-nitrosothiols are long lived bioactive materials that duplicate many of the reactions of free NO. The NA reaction as initially proposed has raised concerns from several perspectives. The affinity of metHb for nitrite is low and is in fact substantially lower than that for NO. This consideration of affinity suggests that the pathway for the proposed reaction would have to progress via the nitrite reacting with the ferric heme-bound NO (38). It has been shown that the reductive nitrosylation (RN) reaction where the ferric NO derivative undergoes reduction to the stable “dead-end” ferrous NO derivative can be quite rapid, raising the question as to whether the NA reaction can compete with the RN reaction under physiological conditions (10, 39). The other serious concern is based on the thermodynamics not favoring the reaction in which free N_2_O_3_ is generated (40).

Studies using glassy matrices (41), sol-gels (14, 42), and solutions (14, 42) have provided evidence for the formation of a spectroscopically distinct species that is being proposed as a relatively stable ferrous-N_2_O_3_ intermediate associated with the NA reaction. This species could in principle preserve NO bioactivity in a manner analogous to that proposed for the *S*-nitrosothiol derivative of HbA (SNOHb) (43–46). The spectroscopic signature of this proposed intermediate has been used to provide evidence both that the NA reaction can proceed with either nitrite or NO binding first to the ferric heme and that the reactivity of the nitrite for ferric heme-bound NO is much higher than anticipated based on its low binding affinity for free ferric heme (14). Additionally the study showed that the NA reaction could efficiently compete with the reductive nitrosylation process initiated by the binding of NO to the met heme, resulting in the dead-end ferrous NOHbA (14). Recent studies on HbA indicate that this proposed intermediate is stable in the absence of excess NO and is capable of directly reacting with GSH to yield *S*-nitrosoglutathione (GSNO) in the absence of oxygen (14). These findings all raise the possibility that the NA intermediate might be a significant player in the complex interplay of nitrite, NO, GSH, and hemoglobin. As a consequence, it is of interest to examine the relative efficacy with which LtHb forms the intermediate relative to HbA.

Although there is experimental support for the existence of the NA reaction and for the formation of N_2_O_3_-bound heme NA intermediate, the reactions, existence, and relevance of the NA intermediate are still speculative and controversial. To this end, the current study also sought to further substantiate the existence of the NA intermediate and establish the properties of the NA intermediate in both HbA and LtHb.

The present study compared HbA and LtHb with respect to the following reactions and processes: 1) reduction of the met derivatives by l-cysteine (as well as redox potential measurements of the intact LtHb and its subunits), 2) the nitrite reductase reaction, 3) the ability to form the intermediate associated with the nitrite anhydrase reaction, and 4) the ability of the nitrite anhydrase reaction to compete with reductive nitrosylation. Additionally, the NA “intermediates” of both HbA and LtHb were probed to further test the validity of the N_2_O_3_-heme assignment. EPR spectroscopy was used to differentiate between ferrous NOHb and the proposed NA intermediate, which share similar features in the absorption spectra. The reactivity of the NA intermediate was evaluated with respect to the production of the triazole derivative of the NO probe 4-amino-5-methylamino-2′,7′-difluorescein (DAF-FM) and of the *S*-nitroso derivative of glutathione under oxygen-free conditions. Under oxygen-free conditions, these reactions require the formation of N_2_O_3_ and as a result are a means of testing the assignment for the proposed NA intermediate.

## EXPERIMENTAL PROCEDURES

### Solution Preparation

All commercial reagents were purchased from Sigma in the purest form available.

The oxygenated derivative of hemoglobin A was prepared and obtained as described previously (14, 34, 42). The oxygenated derivative of LtHb was prepared and characterized as described in earlier work (17, 18). All reactions were conducted in 0.05 m Bis-Tris, pH 7.0. The ferric derivative of LtHb was prepared from stock solutions of oxy-LtHb in 0.05 m Bis-Tris, pH 7.0 buffer that were treated with a solution of potassium ferricyanide (K_3_Fe(CN)_6_), which was subsequently removed by spin column, to form the aquomet (ferric) derivative. Deoxy-LtHb was prepared from the met derivative by first purging the solution with argon followed by reduction with a slight excess of dithionite. NO was added to Hb samples in the form of aliquots of solution containing diethylamine NONOate (Sigma) (amine molecules that release NO in a time- and pH-dependent manner) to the argon-purged deoxy derivative to form the Fe^2+^NO derivative and to argon-purged metLtHb to form the Fe^3+^NO derivative. The comparable derivatives of HbA were prepared similarly. The absorption spectra for these derivatives of LtHb and HbA are very similar but with some small wavelength differences. The main UV/visible absorption peak positions for each of these species for both LtHb and HbA are presented in Table 1. The full visible spectra of these species and of a proposed NA intermediate are shown under “Results.” A basis set derived from the spectra of these derivatives was used to determine the distribution of populations contributing to the overall observed spectrum at a given time point during the time course of the different NO/nitrite-related LtHb and HbA reactions described below.

**TABLE 1:** **Peak positions for Hb absorption bands (nm)**

Ligation state*^a^*	HbA			LtHb		
	Sôret	Q band	Band III	Sôret	Q band	Band III
Oxy	415	541, 577		418	541, 576	
Deoxy	430	555	757	429	559	762
Aquomet	406	500, 630		396	503, 629	
Fe^3+^ nitrite	412	536, 567		415	537, 568	
Fe^2+^NO	418	544, 575		417	543, 572	
Fe^3+^NO	417	535, 565		416	534, 564	

*^a^* 0.05 m Bis-Tris, pH 7.0.

### Spectral Analysis

All ligation and redox states and reactions were monitored spectroscopically using either a Lambda 2 (PerkinElmer Life Sciences) or an Evolution 300 (Thermo Scientific, Madison, WI) spectrophotometer both to characterize absorption spectra of stable starting populations and to follow the changes in the absorption spectrum of hemoglobin as a function of time once a reaction was initiated.

### Deconvolution of Stable and Time-dependent Absorption Spectra

The absorption spectra of evolving solution samples were repetitively scanned at regular time intervals over extended periods of time. Each spectrum was deconvoluted using a basis set consisting of Fe^2+^NO, Fe^3+^NO_2_^−^, aquomet, and Fe^3+^NO, which were prepared individually as described above. Spectral data were deconvoluted using a program in Mathcad v.14.0 (PTC Inc., Needham, MA) as described previously (14, 34) and the basis set as described above. In the case of the NA reaction, the fitting required the addition of another basis set that was assigned to the NA intermediate as described earlier for HbA (14) and under “Results” for LtHb.

### Nitrite Reductase

All solutions and buffers were purged with argon. Deoxy-Hb samples were prepared in an oxygen-free glove box. Nitrite reductase reactions were performed in parallel (HbA and LtHb) experiments using the argon-purged deoxy derivative (∼0.20 mm heme) of either LtHb or HbA in the presence of 1 mm dithionite to which was added aliquots of solutions of nitrite prepared in argon-purged buffer. The ratio of nitrite to heme was varied over a range of 0.5:1 to 10:1. Changes in the deoxy Sôret 430 nm band were recorded as a function of time. This choice is based on the relatively large extinction coefficient for deoxy-Hb at this wavelength (133,000 m^−1^ cm^−1^) compared with NOHb (∼8400 m^−1^ cm^−1^) and metHb (∼4000 m^−1^ cm^−1^). Under these conditions, the nitrite reductase reaction is modified in that the met(Hb^+^) product generated from the nitrite reductase reaction is reduced back to deoxy-Hb by the excess dithionite. The deoxy-Hb binds NO, yielding the ferrous NO derivative of Hb (NOHb). As a result, the monitored reaction consists overwhelmingly of the loss of deoxy-Hb and the formation of NOHb. The initial linear portion of the curve was used to calculate the initial rates using three or more trials. Exponential rates were calculated using the decay curve with a program in Origin 8.5 (Originlab, North Hampton, MA). Products were verified through full UV/visible absorption spectra generated after the 430 nm band no longer changed. Under the conditions used for these measurements, the kinetic traces for HbA only reflect the T state kinetics. In our previous work, we showed traces that manifest the initial T state population switching over to the faster reacting R state population (31, 34).

### Reductive Nitrosylation

All solutions and buffers were purged with argon. Samples of aquometHb were prepared in an oxygen-free glove box. Reductive nitrosylation experiments on LtHb and HbA were conducted in parallel by adding an aliquot of diethylamine NONOate (amine molecules that release NO in a time- and pH-dependent manner) to a solution of the argon-purged ferric derivatives (0.30 mm heme) of HbA or LtHb. Evolution of the sample was monitored by scanning the complete spectrum from 350 to 850 nm every 5 min at a scan rate of 240 nm/min. The resulting spectra were deconvoluted as described below using the basis set established above to establish the fraction of each contributing species as a function of time.

### Nitrite Anhydrase

All solutions and buffers were purged with argon. Aliquots of diethylamine NONOate were added to the ferric derivative of the protein in 0.05 m Bis-Tris, pH 7.0 (∼0.30 mm heme) in the presence of varying levels of initially added nitrite (starting at 1:1 heme up to an excess of nitrite of 30 mm). The evolution of hemoglobin populations was followed by monitoring spectral changes as a function of time as for reductive nitrosylation. The data were deconvoluted as described above using the basis set established above for LtHb and HbA. In some cases, NONOates were added in small incremental aliquots to samples saturated with nitrite (30 mm) to deliver NO more slowly to the ferric nitrite complex.

### Reduction by l-Cys

Samples were purged with argon as were all buffer solutions and were prepared in an oxygen-free glove box. Reactions were performed anaerobically. A stock solution of reduced l-Cys was prepared in argon-purged buffer. A 2 mm aliquot of l-Cys was added to a ferric solution (0.30 mm heme) of the protein. Absorption spectra were scanned at regular intervals. Data were analyzed by recording the change in the 630 nm band as a function of time. In other experiments, l-Cys was used in conjunction with nitrite. The l-Cys was used to slowly reduce the ferric derivative to deoxy-Hb, which in the presence of nitrite results in the slow production of NO and metHb through the NR reaction. This approach allows for the slow production of NO in the presence of nitrite and metHb.

### Redox Properties of LtHb

#### 

##### Autoxidation Studies

Continuous spectra were recorded from 700 to 500 nm using a double beam spectrophotometer (Lambda 20, PerkinElmer Life Sciences) at specified time intervals. All spectral determinations were carried out at 37 °C. The rate of metHb formation was established by the changes in the *A*_576_/*A*_540_ ratio. This method of analysis eliminates light scattering effects.

##### Chemical Oxidation

The traditional mode of chemical oxidation for Hb is potassium ferricyanide. Rates of oxidation of Hbs were determined as described for the autoxidation studies using fixed concentrations of potassium ferricyanide.

##### Redox Potential

Hb redox potential was determined by spectroelectrochemical analysis. The determination of the formal reduction potentials (*E*^0^) for each Hb was carried out using a modified, optically transparent electrochemical cell. An electrochemical cell was constructed based on a previous method (47). Briefly, the spectroelectrochemical experiments were carried out in an anaerobic air-tight optically transparent electrochemical cell with a 1-cm-path length cuvette. A three-electrode system inside the cell was used to apply the potential and read currents. The electrical configuration included two small meshes of platinum connected with platinum wires placed inside walls of the cells (working and working sense electrodes) and Ag/AgCl reference electrode (ee009, Cypress Systems, Lawrence, KS). The electrochemical cell was purged with nitrogen for 10 min prior to injecting protein samples. After the Hb solution was introduced into the cell, the sample was maintained in the deoxy-Hb state by fluxing nitrogen for 1 h. The electrochemical cell was placed in the cell holder in the sample compartment of a spectrophotometer (Lambda 20). Potential control of the three-electrode system was powered by a potentiostat/galvanostat (Model 273A, EG&G Princeton Applied Research, Princeton, NJ) for cyclic voltammetric studies. All potentials were measured relative to the Ag/AgCl electrode. Spectroelectrochemistry measurements were carried out at 430 nm for deoxy-Hb and 406 nm ferric (met)Hb. The potential of the working electrode was held at a positive potential (+200 mV or above depending on the Hb sample) to oxidize all Hb to metHb and the absorbance was set at 0. Next, the potential was reduced to the potential where the amount of reduced material was about 5% (typically by 20 mV) and held until the absorbance did not change (typically 5 min). The final potential change was made from a potential where more than 95% of the material was reduced (typically between −200 and −500 mV). At this negative potential, all Hb solutions were in the reduced state. The absorbance was taken as the total absorbance of the Hb in the deoxy-Hb state. The spectroelectrochemical process was repeated in the oxidative direction. In this case, the initial potential was typically about −500 mV, then the potential was taken to about −200 mV, and the procedure continued in reverse from that described for the reductive direction. Hexaammineruthenium(III) chloride (Ru(NH_3_)_6_Cl_3_; Sigma-Aldrich) was used as a mediator for the electrochemically reversible process in the oxidative and reductive directions. The *E*_1/2_ of the Ru(NH_3_)_6_Cl_3_ is −150 mV at a platinum electrode in the electrochemical cell containing 0.05 m MOPS, 0.20 m KCl, pH 7.10. The Hb oxidation rate was slower than the reduction rate.

The absorbances of the Hb at various potential values were converted to a ratio of oxidized Hb form to reduced Hb form using Equation 1 where *A*_Tred-430_ is the absorbance at 430 nm at the potential where Hb was totally reduced, *A*_Toxi-430_ is the absorbance at 430 at the potential where Hb was totally oxidized, and *A_E_*_-430_ is the absorbance at 430 nm at each potential.

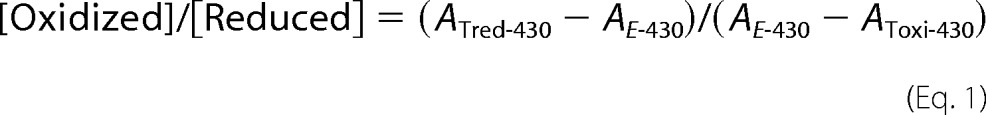
 Because the totally oxidized Hb was set to 0 (*A*_Toxi-430_ = 0), Equation 1 simplifies to Equation 2.

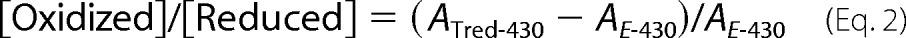
 The log of this ratio when used to plot the function of potential constitutes a Nernst plot where *E* is the potential of the working platinum electrode controlled by the potentiostat, *E*^0^ is the reduction potential (log[oxidized]/[reduced] = 0), *n* is the slope, and 58.1 is the value of *RT/F* at 20 °C.

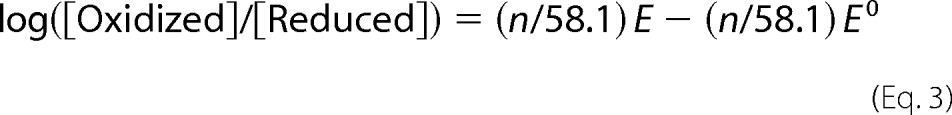
 For a complex redox system like the LtHb, the formal redox potential is difficult to define. Therefore, we define the *E*^0^ value as the value of *E*^0^ at half-oxidation as is done for the case of non-ideal Nernstian behavior (48). For a non-interactive system like myoglobin, the Nernst plot is linear, and the slope *n* is indicative of the number of electrons transferred. Thus *n* would be expected to be unity for Mb. The complex nature of the LtHb redox system precludes the determination of electrons transferred stoichiometrically. For the LtHb, the Nernst plot is only linear over a small range, and the slope *n*, which is not constant, is indicative of interaction and therefore will not correspond to the number of electrons transferred (48). Conditions for all redox measurements were 0.25 mm LtHb, 100 mm MOPS buffer, pH 7, 0.5 mm Ru(NH_3_)_6_Cl_3_ mediator, 200 mm NaCl, 20 °C.

### Data Analysis

The absorbances of the Hb at various potential values were converted to a ratio of [oxidized form]/[reduced form] by using Equation 2 where *A*_Tred-430_ is the absorbance at 430 nm when the Hb was totally reduced, *A*_Toxi-430_ is the absorbance at 430 when the Hb was totally oxidized, and *A_E_*_-430_ is the absorbance at 430 nm at each potential. Because the absorbance of the [oxidized form]/[reduced form] = (*A*_Tred-430_ − *A_E_*_-430_)/(*A_E_*_-430_ − *A*_Toxi-430_) (Equation 1), totally oxidized hemoglobin was set to 0 (*A*_Toxi-430_ = 0), and Equation 1 simplifies to Equation 2: [oxidized form]/[reduced form] = (*A*_Tred-430_ − *A_E_*_-430_)/*A_E_*_-430_. The log of this ratio plotted as a function of potential constitutes a Nernst plot as expressed in Equation 3 where *E* is the potential of the working electrode controlled by the potentiostat and *E*^0^ is log[oxidized]/[reduced] = (*n*/58.1)*E* − (*n*/58.1)*E*^0^. The reduction potential is reported when log[oxidized/reduced] = 0, and the slope × 58.1 corresponds to the number of electrodes transferred based on the Nernst equation. 58.1 is the value of *RT*/*F* at 20 °C.

### Preparation of LtHb Subunits for Redox Potential Measurements: LtHb Fractionation

Preparation of the subunits of *Lumbricus* Hb followed methods described previously (49). Briefly, the large subunits were isolated by dialysis of hemoglobin solution against 0.1 m sodium phosphate buffer, pH 6.8. Then a sample of 20 mg in 0.5 ml was applied to a column of Sephadex G-150 and eluted with the pH 6.8 buffer. The small subunits were prepared by dialysis of a hemoglobin solution against 0.1 m sodium carbonate buffer, pH 10 for 2 h. Then a sample of 15 mg in 0.3 ml was applied to a column of Sephadex G-75 and eluted with the pH 10 buffer. Flow rates of 1–1.5 ml/h were used, and 0.25-ml fractions were collected and read at 280 nm.

### EPR Comparison of the Ferrous Nitrosyl Derivatives of HbA and LtHb with NA Intermediates from HbA and LtHb

Given the similarity between the UV/visible spectra of ferrous NOHb derivatives of HbA and LtHb and their respective spectra attributable to the proposed NA intermediate, it was essential to establish whether the two derivatives from each protein exhibited different EPR signals. Whereas the ferrous NO derivative has a strong EPR signal derived from the free radical nature of the ferrous heme-bound NO, the proposed assignment for the NA intermediate, *i.e.* a ferrous heme-bound N_2_O_3_, should not be EPR-active.

#### 

##### Preparation of EPR Samples

All solutions of protein and salts were prepared using argon-purged buffer. All samples were prepared in an oxygen-free glove box. A stock solution of metHbA (0.35 mm in heme) was prepared in 0.05 m Bis-Tris, pH 7.0. This solution was used to generate Fe^2+^NO, Fe^3+^NO, and Fe^3+^Hb as standards for the EPR measurements. An aliquot of this solution was also used to prepare Fe^3+^ nitrite. The NA intermediate was prepared by adding 10–30 mm nitrite to an aliquot of metHbA followed by ∼0.115 mm NONOate. The sample was allowed to evolve for 1 h until the intermediate spectrum was observed. At that point, 15% glycerol was added, and the sample was frozen in liquid nitrogen. A solution of metLtHb (0.3 mm in heme) was prepared similarly in 0.05 m Bis-Tris, pH 7.0. This solution was used to generate Fe^2+^NO, Fe^3+^NO, Fe^3+^ nitrite, and Fe^3+^Hb. To prepare the intermediate, 30 mm nitrite was added to an aliquot of the solution. Varying amounts of NONOates were added to individual solutions of the met nitrite. Each solution was allowed to evolve for about 1 h until the intermediate spectrum was observed. At that point, 15% glycerol was added, and the samples were frozen in liquid nitrogen. In all cases, the distribution of contributing Hb derivatives was determined as described above using the deconvolution of the absorption spectrum of the sample at the point at which it is frozen and made ready for the EPR measurement.

##### EPR Measurements

X-band (9-GHz) EPR measurements were made using a Varian E-112 spectrometer interfaced to a PC using custom written software. Samples were pipetted into 4-mm quartz EPR tubes and frozen over a bath of liquid nitrogen. A finger Dewar filled with liquid nitrogen and inserted into the TE_102_ EPR cavity maintained the sample at 77 K throughout the measurements. The field was calibrated using a standard sample of manganese doped in MgO (50). Spectrometer parameters used to acquire spectra are listed in figure legends.

### DAF-FM as a Probe of the NA Intermediate

The fluorescence and absorption spectra of diaminofluoresceins such as DAF-FM (51, 52) undergo a substantial change when the triazole derivative is formed (51–56). The fluorescence emission increases dramatically, and the peak position shifts several nanometers from 508 nm to as high as 515 nm. Similarly, the absorption peak in the visible region also shifts from 488 to 496 nm upon formation of the triazole derivative. These changes are routinely used to detect NO production, but oxygen is required to react with the NO to form N_2_O_3_, which is required to produce the triazole. NO by itself is not capable of generating the triazole derivative. In this study carried out under oxygen-free conditions, the spectroscopic changes were used to provide additional evidence that the NA intermediate is a heme-bound N_2_O_3_ derivative. A comparison under oxygen-free conditions of the spectroscopic changes in the absorption and fluorescence spectra of DAF-FM for the ferrous NO derivatives and for the NA intermediate was made for HbA and LtHb. The comparison was in effect made by adding NO (NONOates) to samples of metHbs (∼0.30 mm) with and without additionally added nitrite and allowing the samples to evolve for varying time intervals before adding DAF-FM (5 μm from a stock solution in DMSO) under oxygen-free conditions. The sample with NONOates but without added nitrite was prepared in anticipation of it evolving through reductive nitrosylation to the ferrous NOHb derivative, whereas the sample with both NONOates and nitrite was anticipated to yield a substantial population of the proposed NA intermediate. Absorption spectroscopy was used to confirm the formation of the two derivatives of Hb. Under anaerobic conditions, the NA intermediate was expected to be much more effective compared with the ferrous NOHb sample in generating the triazole derivative of the DAF-FM. The samples containing DAF-FM were excited using 475 nm rather than the peak wavelength 495 nm to better observe changes in the emission spectrum.

### HPLC Measurements

HPLC was performed on a Restek C_18_ 5-μm reverse phase column (250 × 4.6 mm; Restek Corp., Bellefonte, PA). The running buffer was 10 mm KPO_4_ buffer, 10 mm tetrabutylammonium bisulfate, 5% acetonitrile, pH 7.0 at a flow rate of 1 ml/min. Samples were run using a Shimadzu HPLC equipped with Class VP V. 7.4 software (Shimadzu, Kyoto, Japan). Elution times and UV-visible spectra were compared with authentic standards of nitrite, GSNO, glutathione disulfide, and GSH (Sigma) prepared and run using the same elution buffer.

All solutions of protein and salts were prepared using argon-purged buffer. All samples were prepared in an oxygen-free glove box. A stock solution of metLtHb (0.3 mm in heme) in 0.05 m Bis-Tris, pH 7.0 was prepared and divided into two samples of equivalent volume. One-half was treated with both NONOates and nitrite to produce the NA intermediate, and the second sample was treated with NONOates alone and allowed to evolve into the ferrous NO derivative.

#### 

##### LtHb NA Intermediate

The sample being evaluated as the LtHb NA intermediate was prepared by first adding nitrite to a final concentration of 30 mm followed by NONOates (0.115 mm). The sample was monitored by absorption spectroscopy as it evolved. When the intermediate spectrum was observed, the buffer was removed by spin column and replaced with argon-purged buffer to which was added 5 mm GSH and 0.5 mm diethylenetriaminepentaacetic acid, the copper chelator. The sample was incubated for 1 h after which the buffer was again removed by spin column, and the eluent was run on an HPLC column.

##### Ferrous LtHbNO Control

The second solution was treated only with NONOates in the identical manner as described above for NA intermediate sample but without any added nitrite. The metLtHb + NONOate sample was allowed to evolve to Fe^2+^NO as determined by absorption spectroscopy. The buffer was removed by spin column and replaced with 5 mm GSH and diethylenetriaminepentaacetic acid in argon-purged buffer. The sample was allowed to evolve for 1 h after which the buffer was removed, and the eluent was run on the HPLC column.

## RESULTS

### 

#### 

##### Nitrite Reductase

**FIGURE 1.**
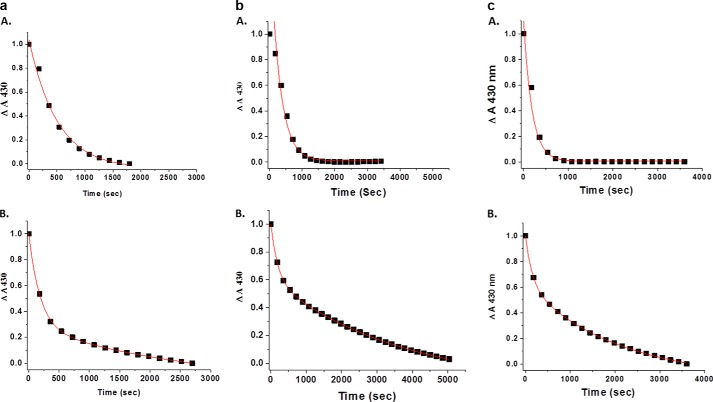
**A comparison between HbA (traces *A*) and LtHb (traces *B*) of the rate of nitrite reductase at pH 7.0 monitored as the decay of the 430 nm Sôret band as a function of the nitrite to heme ratio: 1:1 (*a*), 2:1 (*b*), and 5:1 (*c*).** The heme concentration in all cases was ∼0.20 mm. The *red lines* represent fits to the data using Origin 8.5 as described under “Experimental Procedures.” The traces were all normalized to better expose the comparison of the kinetics.

Fig. 1 shows a comparison between HbA and LtHb with respect to the normalized nitrite-induced decay of the deoxyheme population as a function of the ratio of added nitrite to heme while keeping the heme concentrations relatively fixed. It can be seen that at all ratios of nitrite to heme LtHb appears to exhibit two distinct phases, whereas under these conditions only one phase is observed for HbA. Exponential rates were calculated using both single and double exponential fitting algorithms associated with the program in Origin 8.5 (*y* = *y*_0_ + *Ae*^−*kt*^ or *y* = *y*_0_ + *A*_1_*e*^−*kt*1^ + *A*_2_*e*^−*kt*2^). For HbA, a single exponential algorithm fit extremely well; however, for LtHb, the single exponential fit was poor, whereas the double exponential fit was much better. The goodness of fit for each of the kinetic curves was determined by comparing both the *R*^2^ value and the χ^2^ value for each equation used. Fits were chosen that had *R*^2^ values closest to 1 (0.97–0.99) or χ^2^ values of 10^−4^ or less.

Under these conditions, only the T state contributes to the observed trace for HbA (31, 34). LtHb does not undergo any R/T allosteric transitions; hence the two distinct phases for LtHb are likely to be attributable to different heme populations. The rates displayed in Tables 2 and 3 show that the faster initial components of the decay curve for LtHb are also consistently slower than the initial rates of decay observed for HbA. The slow phase is consistently much slower than the overall decay seen for HbA. The fraction of the total LtHb population undergoing the faster initial decay appears to be consistently below 20% as seen from Table 2. Despite initiating these measurements at the exact moment the reagents were mixed, there is still some scatter with respect to the fraction of fast and slow decay components that is attributable to the difference in the exact start times for the measurements. The intent of these data is to demonstrate the difference between the proteins. Detailed evaluation of rates and rate constants requires a more comprehensive study that is planned for the future.

**TABLE 2:** **Deoxy-LtHb + nitrite kinetic rates in 0.05 m Bis-Tris, pH 7.0 (μm/s)**

(LtHb deoxy) + NO_2_^−^	Percent fast phase	Rate	
		Fast phase*^a^*	Slow phase*^b^*
300:1 (0.120 mm)	10	0.143	0.035
5:1 (0.260 mm)	20	0.0485 ± 0.003	0.012 ± 0.001
2:1 (0.184 mm)	9	0.018 ± 0.002	0.0038 ± 0.0012
1:1 (0.227 mm)	17	0.0635 ± 0.011	0.0036 ± 0.0009
0.5:1 (0.169 mm)	13	0.046 ± 0.008	0.0028 ± 0.0006

*^a^* Calculated using an exponential fit (*y* = *y*_0_ + *Ae*^−*kt*^) to the slope, corrected for the concentration.

*^b^* Calculated by using a linear fit (*y* = *mx* + *b*) in Origin 8.5 to the last part of the data.

**TABLE 3:** **Deoxy-HbA + nitrite kinetic rates in 0.05 m Bis-Tris, pH 7.0**

(HbA deoxy) + NO_2_^−^	Initial rate*^a^*	Rate*^b^*
	μ*m/s*	*1/s*
5:1 (0.304 mm)	0.377	0.488
5:1 (0.248 mm)	0.602	0.403
2:1 (0.291 mm)	0.159	0.202
1:1 (0.230 mm)	0.135	0.148
0.5:1 (0.311 mm)-0	0.09	0.062

*^a^* Calculated by using a linear fit (*y* = *mx* + *b*) in Origin 8.5 to the initial slope of the data.

*^b^* Calculated using Origin 8.5 (*y* = *A*_0_*e*^−*kt*^) to the slope, normalized to the concentration.

##### Redox Potentials

**FIGURE 2.**
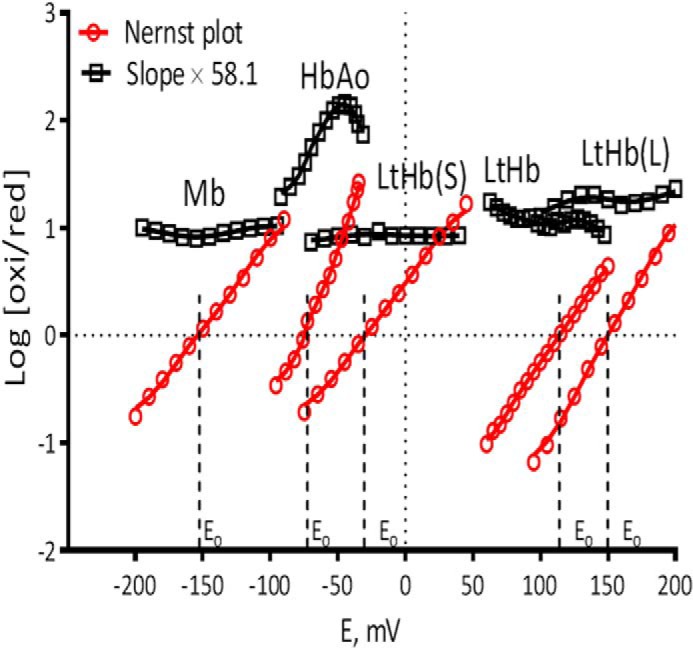
**A comparison of the Nernst titrations for LtHb, the large and smaller LtHb subunits, HbA, and myoglobin (horse heart).** See “Experimental Procedures” for a full description of how the curves were generated. *oxi*, oxidized; *red*, reduced.

Fig. 2 shows both the Nernst plot redox titrations and the corresponding slopes for LtHb, LtHb subunits, HbA, and Mb. Table 4 contains the measured redox potentials of Mb, HbA, LtHb, and two LtHb subunits. The ordering of the redox potentials follows previously published results with Mb having the lowest value and LtHb having the highest (27, 57). The two LtHb subunits have dramatically different values with the larger subunits having an extremely high positive value and the smaller subunit having a negative value but not as negative as either HbA or Mb.

**TABLE 4:** **Redox potential (spectroelectrochemical) at T = 20 °C, 200 mm NaCl**

	*E*^0^
	*mV*
Myoglobin	−154 ± 8
HbAo	−73 ± 5
LtHb	114 ± 9
LtHb (small subunits)	−30 ± 2
LtHb (large subunits)	−150 ± 8

##### Reduction of metHb by l-Cysteine

**FIGURE 3.**
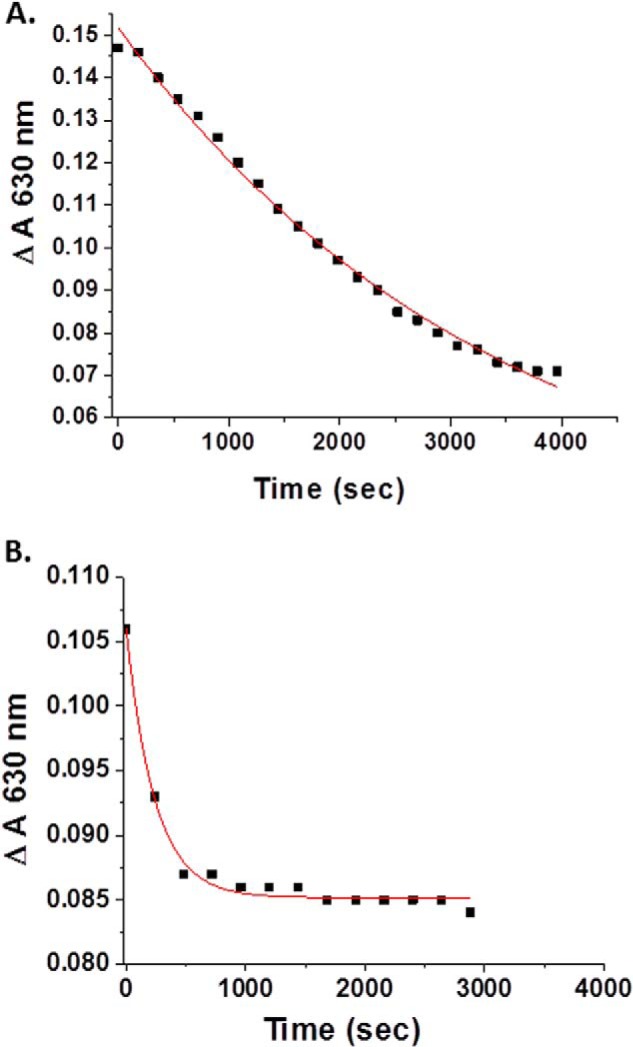
**A comparison of the rate of reduction of the aquomet derivative of HbA (*A*) and LtHb (*B*) by l-cysteine at pH 7.0.** The reduction was followed by monitoring the change in absorbance of the 630 nm band of HbA (*A*) and LtHb (*B*). The *red lines* represent fits to the data using Origin 8.5. Protein concentration was 0.308 in *A* and 0.304 in *B*. l-Cys concentration was 2 mm in both cases.

*In vivo* reductants such as l-Cys are of potential relevance in mediating redox activity of Hbs (58). Fig. 3 shows the comparison between HbA and LtHb with respect to the rate of l-Cys-induced reduction of the aquomet derivatives of the two Hbs. The rate for LtHb is substantially faster than for HbA, consistent with LtHb having the higher redox potential. An analysis of the fraction of the total population undergoing reduction revealed that less than 20% of the population undergoes the reduction even in the presence of excess l-Cys. For HbA under the same conditions, 50% of the initial met population undergoes reduction, suggestive of an αβ chain difference in reactivity with respect to l-Cys-induced reduction. These percentages are shown in Table 5.

**TABLE 5:** **Percentage of aquomet reduced by l-Cys**

l-Cys reduction*^a^*	HbA	LtHb
	%	%
2 mm	50	16
5 mm	50	17

*^a^* 0.30 mm aquomet (heme), 0.05 m Bis-Tris, pH 7.0.

##### Reductive Nitrosylation

**FIGURE 4.**
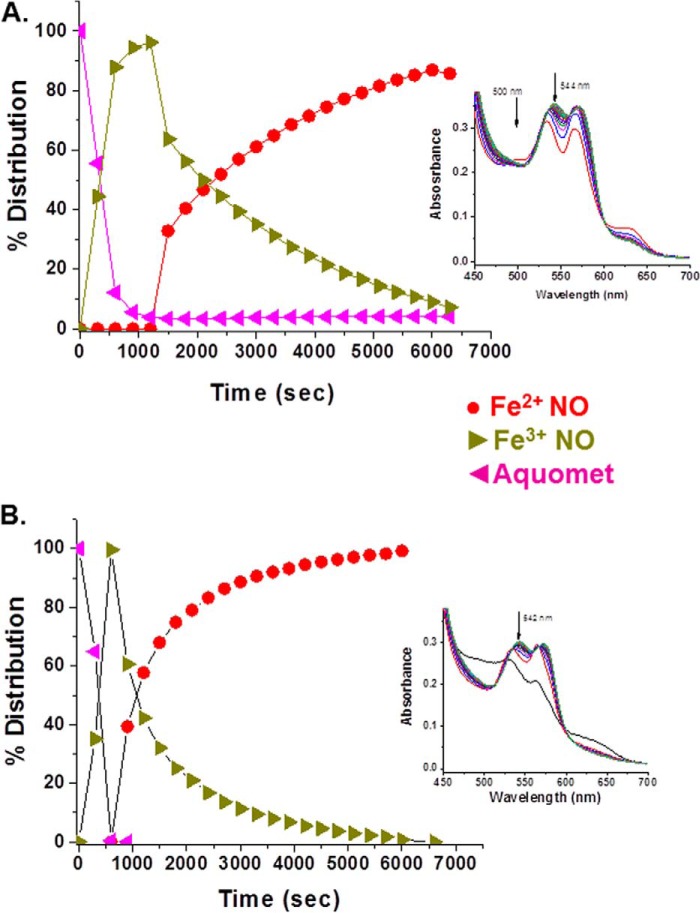
**A comparison of the rate and efficacy of reductive nitrosylation between HbA (*A*) and LtHb (*B*) upon adding an aliquot of NO (1:1, NONOate:heme) to the metHb derivatives at pH 7.** The populations shown were derived from deconvoluting the evolving absorption spectra subsequent to the addition of the NONOates to the Hb solutions at time 0. *Insets* show a representative series of visible absorption bands generated during the course of the reactions. The fittings of these absorption bands were used to derive the evolution of the populations. The time sequence for the shown spectra starts from the *bottom* spectrum.

Fig. 4 compares HbA and LtHb with respect to the rate of reduction of metHb with concomitant formation of the ferrous NO derivative by the addition of stoichiometric amounts of NO (supplied via NONOates). This figure and most of the subsequent figures show the time evolution of the different populations of Hb derivatives based on fittings (see “Experimental Procedures”) using the series of time-ordered spectra that are shown as an *inset* in each of these figures (the temporal series starts with the *bottom* spectrum). The results show that the RN process is faster for LtHb. This enhanced rate for LtHb was observed under a wide range of added NONOate concentrations (data not shown).

##### Nitrite Anhydrase

**FIGURE 5.**
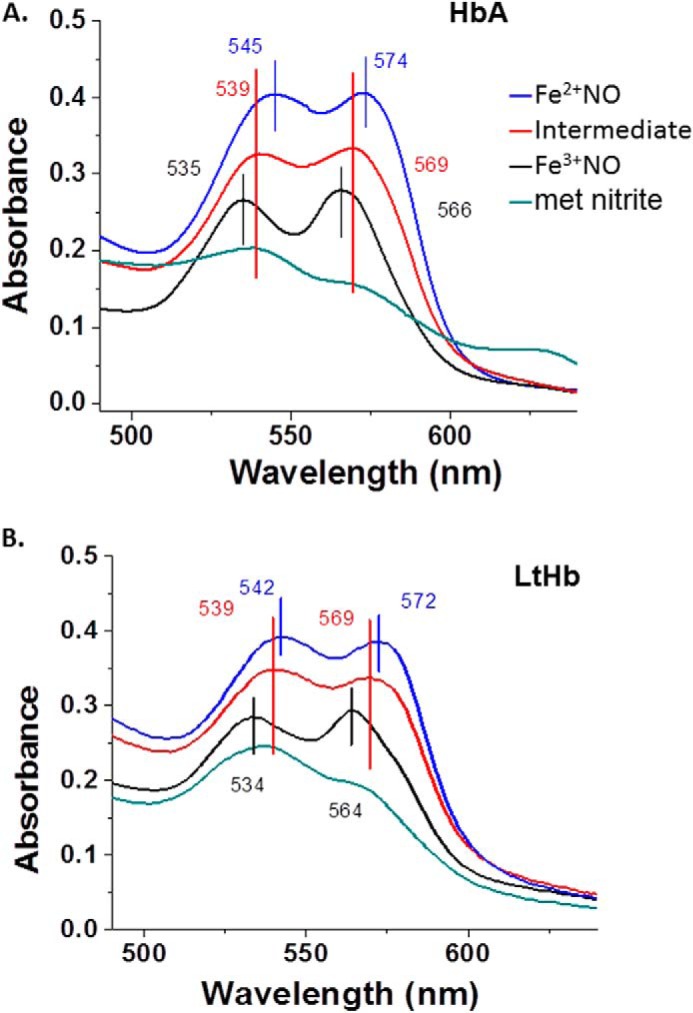
A comparison of the Q band (*i.e.* the major visible heme-associated absorption peak) absorption spectrum of the NA intermediate with the spectra of several stable well known derivatives of HbA (*A*) and LtHb (*B*) at pH 7.

**FIGURE 6.**
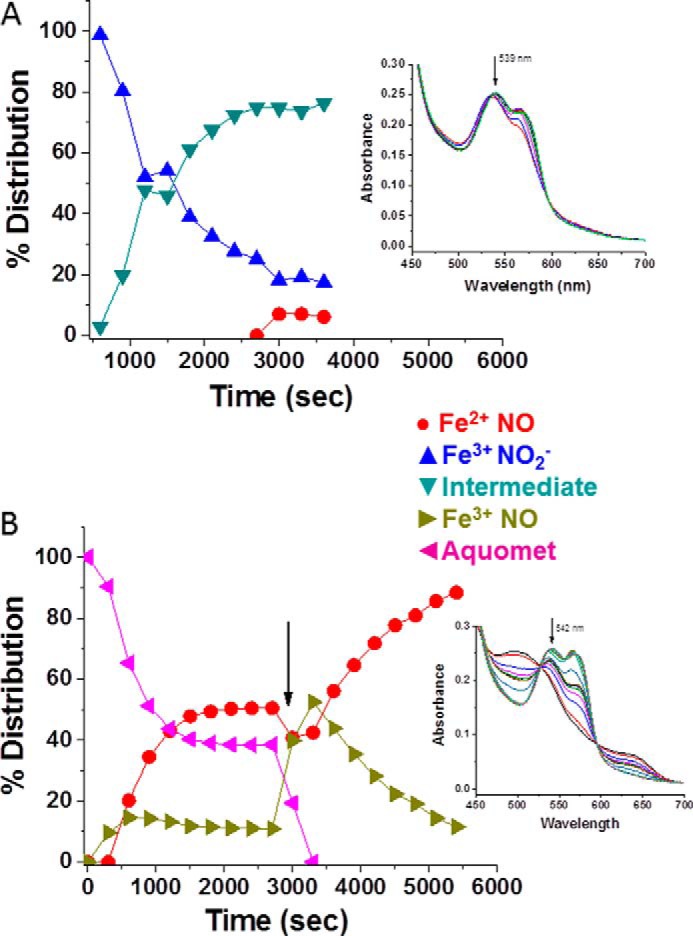
**The evolution of LtHb populations at pH 7 initiated by the addition of NONOates to the ferric derivative of LtHb in the presence (*A*) and absence (*B*) of nitrite.** In both cases, the reaction was initiated with the addition of an aliquot of NONOate-containing solution to a solution of metLtHb (0.25 mm in heme) resulting in a final concentration of 0.075 mm NONOates. The *arrow* in *B* indicates the addition of a second identical aliquot. The metLtHb sample used to generate *A* contained 30 mm nitrite. *Insets* show a representative time sequence of spectra from which the populations were derived.

**FIGURE 7.**
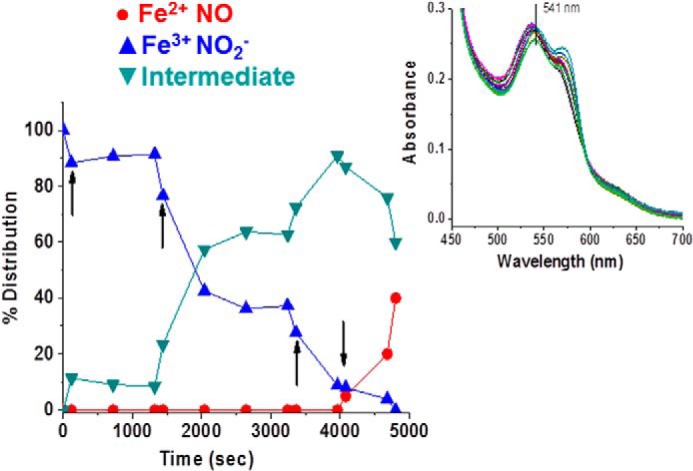
**The evolution of LtHb populations with a sequence of additions of aliquots of NONOates to a sample of metLtHb at pH 7 in the presence of excess nitrite (30 mm).** The *arrows* signify the addition of aliquots of NONOate-containing buffer (0.035 mm/addition). The *inset* shows the time sequence of spectra from which the populations were derived.

**FIGURE 8.**
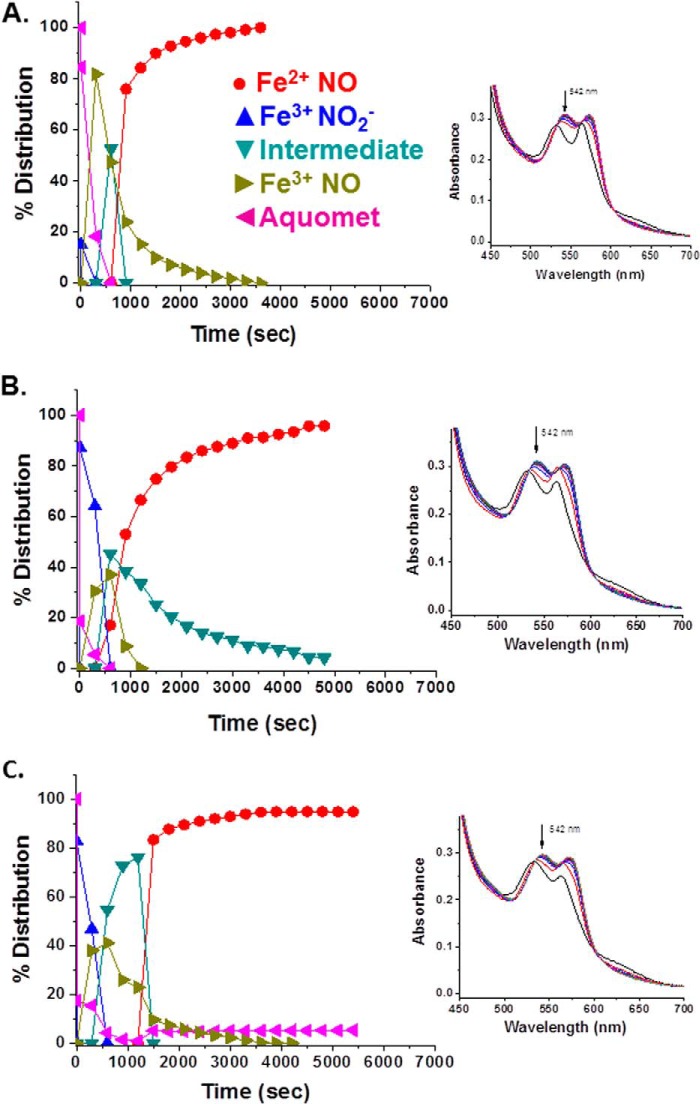
**Evolution of a metLtHb sample (0.29 mm heme) at pH 7 as a function of initial nitrite concentration upon addition of an aliquot of a NONOate solution yielding a final ratio of NONOate to heme of 1:1.** *A*, 1 mm nitrite initially added prior to the addition of NO; *B*, 10 mm nitrite initially added; *C*, 30 mm nitrite initially added. *Insets* show representative time sequences of spectra from which the populations were derived.

**FIGURE 9.**
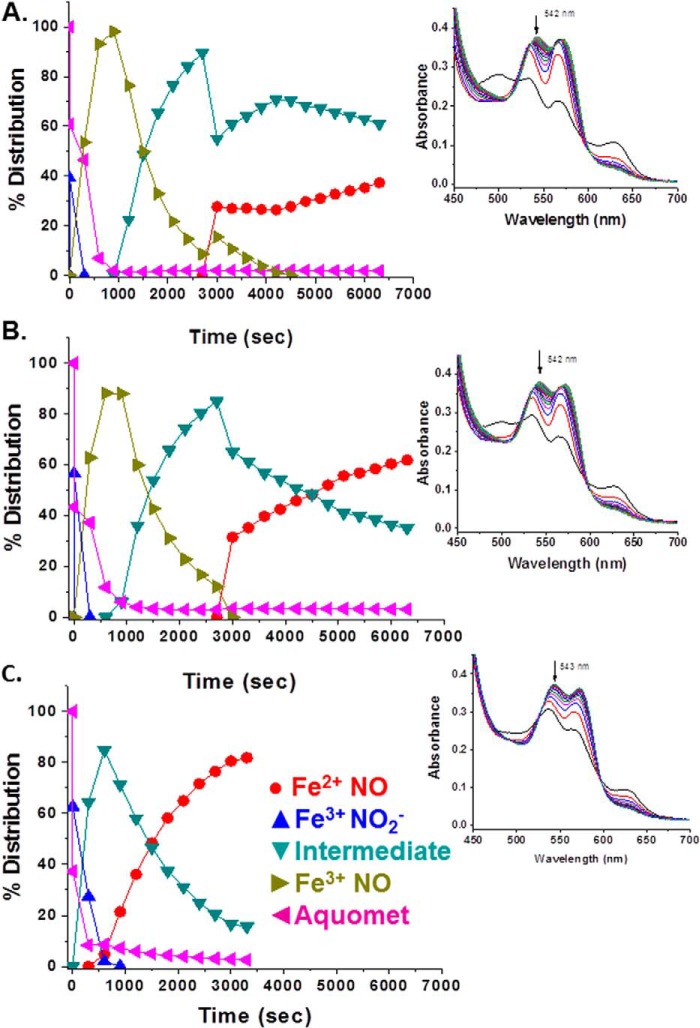
**Evolution of a metHbA sample (0.29 mm heme) at pH 7 as a function of initial nitrite concentration upon addition of an aliquot of a NONOate solution yielding a final ratio of NONOate to heme of 1:1.** *A*, 1:1 nitrite initially added prior to addition of NO; *B*, 1 mm nitrite initially added; *C*, 10 mm nitrite initially added. *Insets* show a representative time sequence of spectra from which the populations were derived.

**FIGURE 10.**
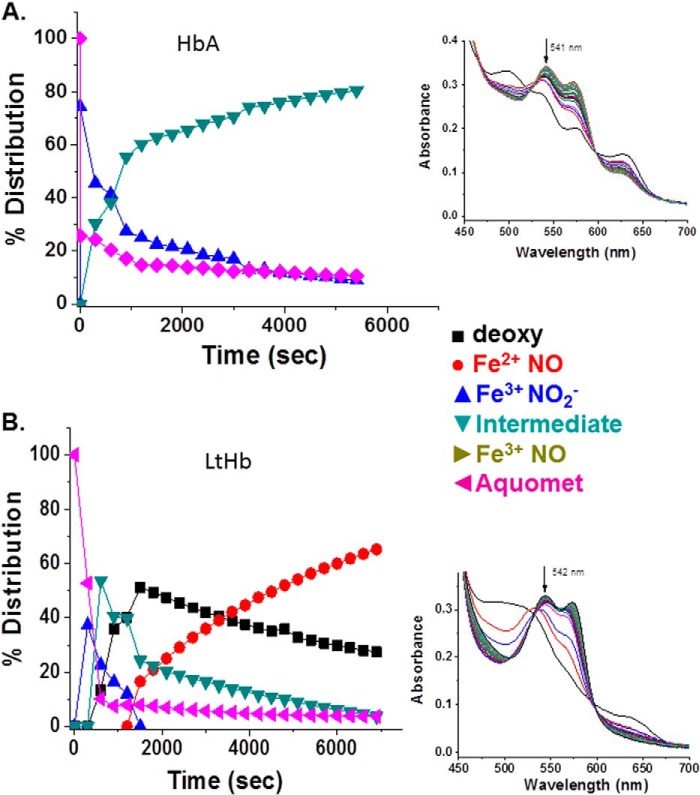
**A comparison of the evolution of the ferric derivatives (0. 3 mm) of HbA (*A*) and LtHb (*B*) in the presence of 4 mm nitrite at pH 7 upon addition 2 mml-cysteine.** *Insets* show a representative time sequence of spectra from which the populations were derived.

Earlier work on HbA demonstrated the formation of a spectroscopically distinct species formed when both NO and nitrite are added to metHbA (14, 34, 41). That species was assigned in those earlier studies as the NA intermediate in which N_2_O_3_ is coordinated to a ferrous heme. It was also shown that the species associated with the NA intermediate can generate *S*-nitrosothiols such as GSNO (14). It was shown based on the formation of this spectroscopically distinct species that for HbA the NA reaction can effectively compete with the RN reaction (14, 34). Here we addressed whether the intermediate can be generated by LtHb and, if so, whether the NA reaction can compete with the RN reaction given the high redox potential and fast rate of RN for LtHb. As was done with HbA, we started with an excess of nitrite (30 mm), thus creating a substantial population of met nitrite for LtHb. The addition of a small amount of NO (via NONOates) resulted in the transformation of the met nitrite spectrum to one that resembles the HbA intermediate. The same transition occurred when low levels of NO were slowly generated by deoxy-LtHb produced by the addition of the reductant l-Cys to an LtHb sample with a large initial population of met nitrite. Fig. 5 compares for both LtHb and HbA the Q band spectra of the intermediate with the Q band spectra from ferric and ferrous NO derivatives and from the met nitrite derivative. Table 6 lists the spectral peaks for all of these NO*_x_*-related species. The spectrum of the NA intermediate was then used as a member of the basis set to fit the evolving populations during the course of reactions in which both NO and nitrite were added or generated. Fig. 6 compares the evolution of populations for metLtHb upon addition of a very low amount of NONOates in the presence (*A*) and absence (*B*) of an excess of nitrite. In the presence of the large excess of nitrite, the addition of the NONOates generates primarily the intermediate, whereas in the absence of nitrite, the addition of the identical low amount of NONOate triggers the RN reaction. It can be seen that at this level of added NO the RN reaction does not go to completion but instead plateaus after a rapid initial buildup of the ferrous NO derivative of LtHb. The addition of a second aliquot does drive the reaction to near completion but at a slower rate with a noticeable buildup of the met NO derivative prior to formation of the ferrous NO derivative. Fig. 7 shows the evolution of an initial population of the met nitrite derivative of LtHb upon addition of very small aliquots of NONOates to a metLtHb sample in the presence of excess nitrite. The pattern is very similar to that reported previously for HbA in that there is a progressive buildup of intermediate with addition of NO until the population of intermediate approaches 80% after which the further addition of NO results in the formation of the ferrous NO derivative. Fig. 8 compares the evolution of the populations for a sample of metLtHb upon addition of a stoichiometric amount of NO (NONOates) as a function of initial nitrite concentration. Fig. 9 shows a comparable series of traces for HbA. In both cases, the fraction of formed intermediate increases with nitrite concentration; however, the fraction of intermediate relative to ferrous NO is consistently much higher for HbA. Fig. 10 shows that under conditions where reduction of metHbA by l-Cys in the presence of 4 mm nitrite results in the progressive buildup of intermediate the comparable set of conditions for metLtHb produces a substantial amount of deoxyheme and ferrous NO heme populations with a much lower intermediate population that decays with time. Similar patterns are seen for lower and higher concentrations of nitrite.

**TABLE 6:** **Peak positions for Hb absorption bands (nm)**

Ligation state*^a^*	HbA		LtHb	
	Sôret	Q band	Sôret	Q band
NA intermediate	417	539, 569	416	539, 570
Fe^2+^NO	418	544, 575	417	542, 572
Fe^3+^NO	417	535, 565	416	534, 564
Fe^3+^ nitrite	412	536, 567	415	537, 568

*^a^* 0.05 m Bis-Tris, pH 7.0.

##### The Anaerobic Response of the DAF-FM Fluorescence and Absorption Spectra to the Presence of the NA Intermediate

**FIGURE 11.**
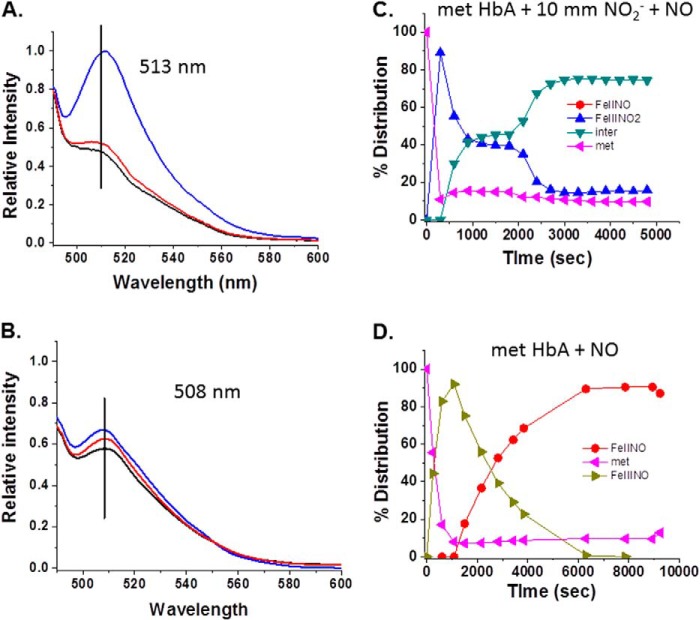
**Changes in the DAF-FM fluorescence emission for HbA solutions.** The increase and the shift in the emission spectrum of DAF-FM as a function of time subsequent to the addition of the fluorophore (5 μm final) to a solution of HbA (0.3 mm) are shown. *A*, metHbA to which was added both nitrite and NONOates to produce the NA intermediate. *Black line*, initial spectrum upon addition; *red line*, change in the emission after 20 min; *blue line*, change in emission after 40 min. *B*, control ferrous NO sample in the absence of nitrite as described under “Experimental Procedures.” *Black line*, initial emission spectrum upon addition of 5 μm DAF added to the protein-NO complex; *red line*, change in the emission after 20 min; *blue line*, change in emission after 40 min. *C*, the evolution of the population distribution profile for the sample prepared to promote formation of the NA intermediate and then evaluated using DAF-FM as described under “Experimental Procedures.” *D*, the evolution of the population distribution profile for the control ferrous NO sample to which DAF-FM was added. *inter*, intermediate.

**FIGURE 12.**
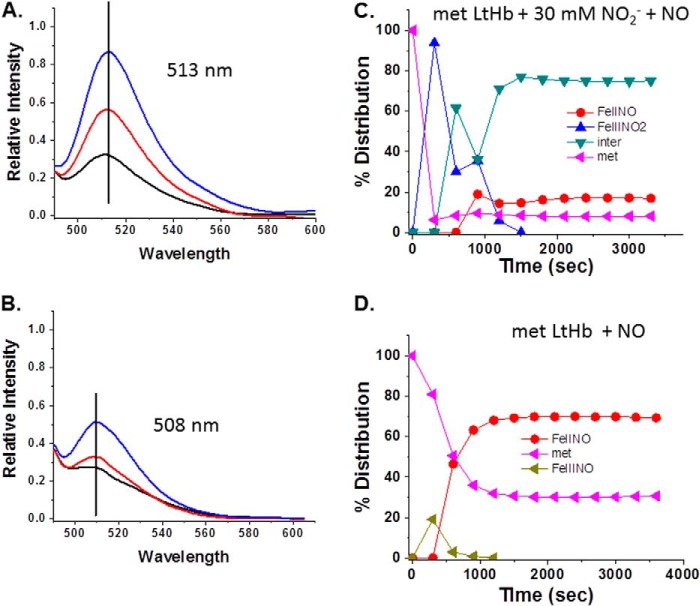
**Changes in the DAF-FM fluorescence emission for LtHb solutions.** The increase and the shift in the emission spectrum of DAF-FM with time when the fluorophore (5 μm final concentration) was added to a solution of LtHb (0.3 mm) are shown. *A*, metLtHb to which was added nitrite and NO. *Black line*, initial emission spectrum upon addition of 5 μm DAF to the LtHb solution; *red line*, change in the emission after 20 min; *blue line*, change in emission after 40 min. *B*, a control ferrous NO sample in the absence of nitrite as described under “Experimental Procedures.” *Black line*, initial emission spectrum upon addition of 5 μm DAF; *red line*, change in the emission after 20 min; *blue line*, change in emission after 40 min. *C*, the evolution of the population distribution profile for the sample prepared to promote the formation of the intermediate used with DAF-FM as described under “Experimental Procedures.” *D*, the comparable evolution of the population distribution profile for the control sample to which DAF-FM was added. *inter*, intermediate.

Figs. 11 and 12 show the evolution of the DAF-FM fluorescence spectra under anaerobic conditions when DAF-FM was added to samples prepared as either the ferrous NO derivative or the NA intermediate of HbA and LtHb. The absorption spectrum was used to establish that in each case the populations were overwhelmingly either ferrous NOHb or the intermediate. For both HbA and LtHb, the increase in fluorescence intensity and the red shift of the fluorescence peak were greater and more rapid for the sample that manifests the absorption spectrum attributable to the NA intermediate (nitrite and NO both added). A similar shift pattern was seen in the absorption maximum of DAF-FM that is also indicative of triazole formation for the samples manifesting the NA intermediate spectrum (not shown).

##### Comparison of the EPR Spectra from NOHb and the NA Intermediate

**FIGURE 13.**
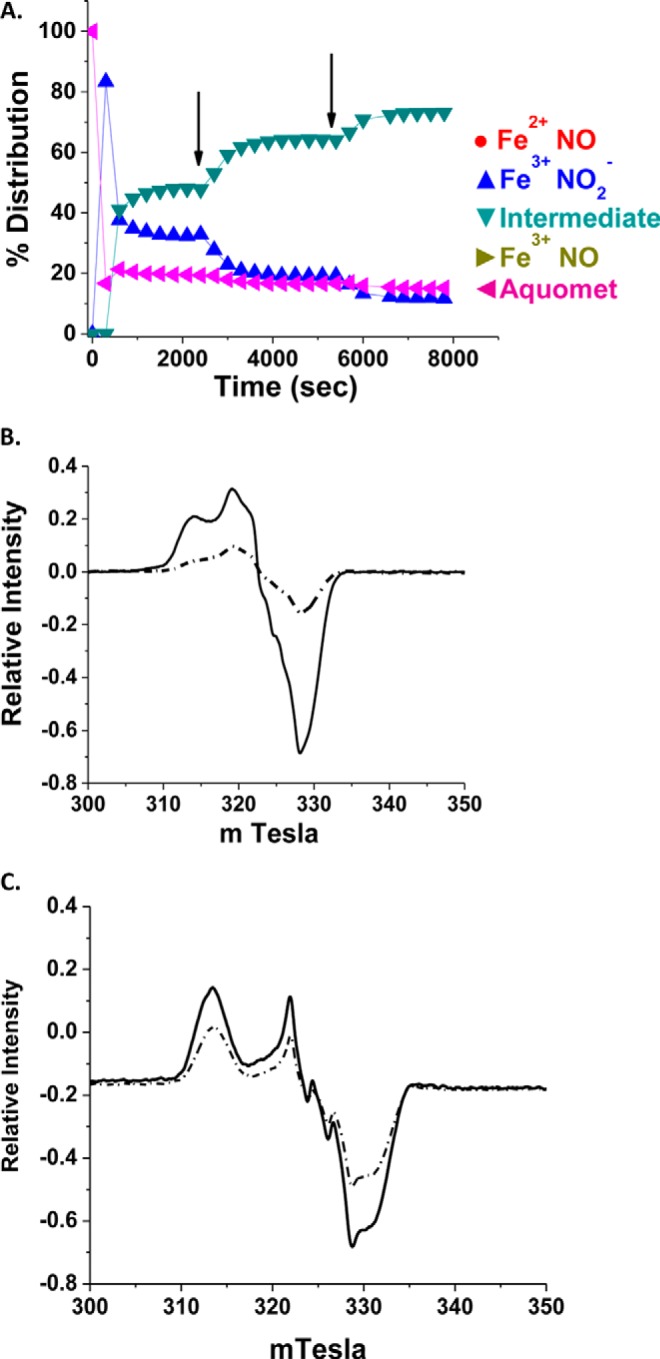
**EPR evidence for a distinct non-ferrous NOHb species.** *A*, a representative trace of the population evolution for an HbA sample (0.302 mm heme) in 0.05 m Bis-Tris, pH 7 to which was added 30 mm NO_2_^−^ followed by an initial aliquot of NONOates (0.067 mm) and two subsequent aliquots of 0.067 mm NONOates (at the two *arrows*) resulting in a total NONOate concentration of 0.201 mm. Once the purported NA intermediate spectrum developed, the sample was then prepared for EPR analysis. The control reference samples did not have nitrite and had sufficient NONOates added to allow the sample to evolve to a close to a fully ferrous NO sample prior to EPR analysis. The comparisons made in the EPR used samples that had the same Hb concentrations for both HbA and LtHb. *B*, EPR spectra (X-band) of Fe^2+^-NO species. *Solid line*, normalized EPR signal in the Fe^2+^NO region for a standard Hb Fe^2+^NO (0.302 mm in heme); *dashed-dotted line*, the normalized EPR signal for the metHbA (0.302 mm in heme) in 0.05 m Bis-Tris, pH 7.0 to which was added both nitrite and NO. EPR instrument parameters for both spectra are as follows: modulation amplitude, 5 gauss; microwave power, 5 milliwatts; receiver gain, 1.25 × 10^3^; microwave frequency, 9.12 GHz; scan time, 2 min; time constant, 0.5 s; number of scans, 4; temperature, 77 K. The spectra were normalized to the intensity of the standard sample. *C*, *solid line*, normalized EPR signal in the Fe^2+^NO region for a standard LtHb Fe^2+^NO (0.30 mm in heme); *dashed dotted line*, the normalized EPR signal for the metLtHb (0.30 mm in heme) prepared with the addition 30 mm NO_2_^−^ and 0.115 mm NONOate.

Fig. 13 compares for both HbA and LtHb the EPR spectrum of populations of fully ferrous NOHb with populations that are ∼70–80% “NA intermediate” based on the population analysis of the absorption just prior to the EPR measurements. The remainder of the population comprises aquometHb, nitrite metHb, and a low level of ferrous NOHb. The NOHbA EPR spectrum is largely consistent with that observed previously for HbA incubated with NO to form six-coordinate Hb(NO)_4_ (59, 60). It is noted that in the spectra presented in Fig. 13*B* the negative peak (trough) in the spectrum has slightly more intensity (∼15%) than would be expected for pure Hb(NO)_4_. We attribute this extra intensity to underlying spectra originating from a residual small percentage of ferric HbA, which would escape detection in the absorption spectrum. Similar spectra have been observed in studies of NO reactions with hemoglobin under heterogeneous conditions (61). The contribution of this residual signal is minimal and does not influence the overall conclusions drawn from the analysis of EPR spectra presented here. The LtHb EPR spectrum (Fig. 13*C*) has a more prominent low field peak as well as partially resolved ^14^N hyperfine splitting. These features indicate structural differences in the heme environment relative to NOHbA with a variation in the ligand arrangement of six-coordinate nitrosyl hemes and/or a contribution from five-coordinate species (59, 60). Further characterization of the EPR spectra from both NOHba and NOLtHb is in progress.

For both HbA and LtHb, the concentration of protein is the same for the NOHb and NA intermediate samples. Thus for both HbA and LtHb comparisons, the EPR signals are derived from samples that are 100% ferrous HbNO (control) and samples that have a 70–80% population of the NA intermediate with the remainder of the population a mixture of met derivatives and ferrous NOHb. It can be seen in both cases that the signal from the composite sample is substantially less than for the fully ferrous NO sample. The residual ferrous NO signals for the HbA and LtHb NA intermediate samples are ∼25 and 50% of the control ferrous NOHb samples, respectively. Varying the percentage of HbNO and NA intermediate (using different concentrations of NONOates and nitrite) produce EPR spectra that scale with the fraction of ferrous NO; *i.e.* the greater the percentage of ferrous NO relative to NA intermediate in the population analysis, the greater is the intensity of the EPR signal. Most significant is that the inclusion of nitrite always lowers the intensity of the EPR signal relative to that from the identical sample to which is added the same amount of NONOates. In all cases, the fraction of metHb is very low compared with the composite population of HbNO and NA intermediate.

##### NA Intermediate of LtHb Can Generate GSNO: HPLC Result

**FIGURE 14.**
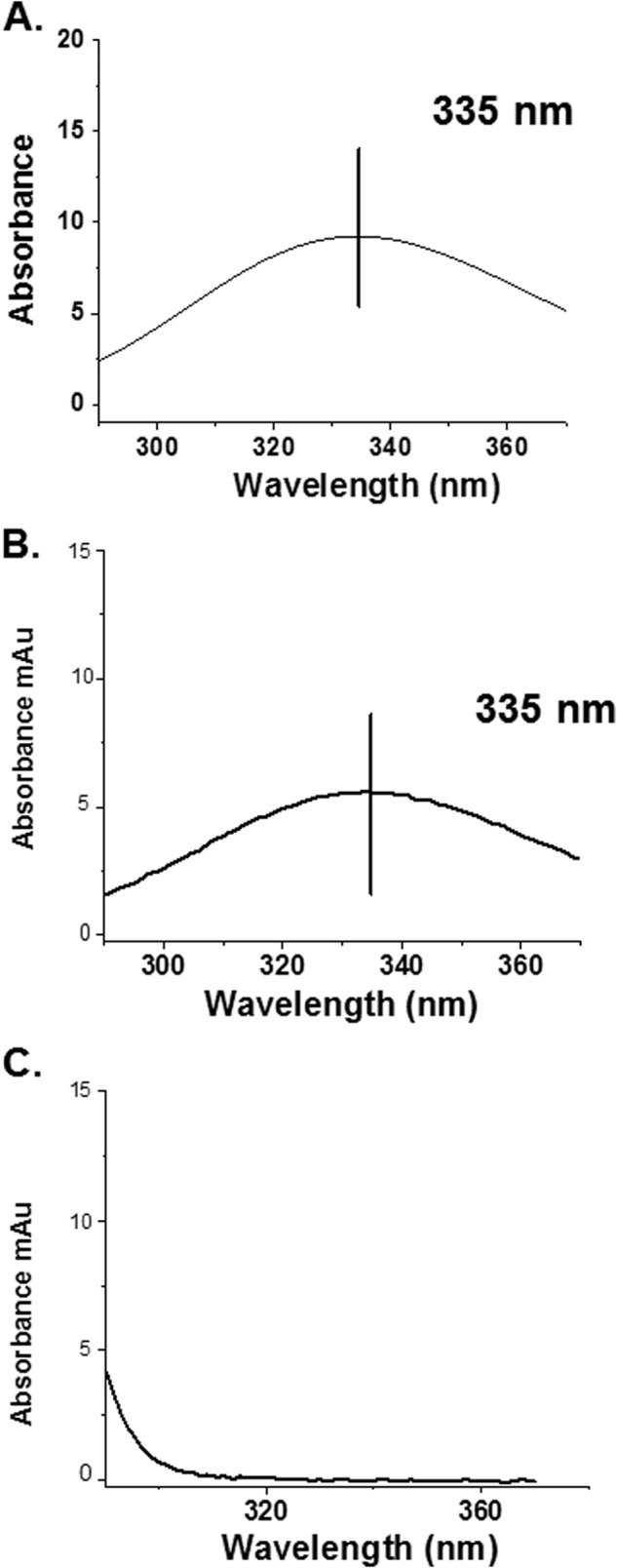
**HPLC evidence of GSNO formation via the NA intermediate of LtHb.** *A*, absorption spectrum recorded on the Shimadzu HPLC of the peak eluting at the retention time for GSNO for the standard solution prepared as described under “Experimental Procedures.” *B*, absorption spectrum recorded on the Shimadzu HPLC for the eluent removed from the LtHb sample prepared (nitrite and NO) to promote the formation of the NA intermediate as describe under “Experimental Procedures.” *C*, absorption spectrum taken for the eluent removed from the control ferrous NOLtHb sample (no nitrite) to which was added GSH (note the difference in the absorption scale). *mAu*, milliabsorbance units.

Fig. 14 compares the absorption spectrum of the HPLC eluent in the region of the observed peak for GSNO for two samples of metLtHb to which was added NONOates with and without nitrite. As described under “Experimental Procedures,” the samples were allowed to evolve to the point where the sample without added nitrite was fully ferrous NOHb and the nitrite-containing sample was predominantly the NA intermediate. At that point, GSH was added, and the samples were treated as described under “Experimental Procedures.” The eluent spectrum from the sample that underwent the full nitrite plus NO protocol shows the same eluent spectrum as the GSNO standard (plus substantial residual GSH). No such spectrum is observed for the eluent from the control sample without nitrite. This observation is indicative of GSNO formation occurring under anoxic conditions for the sample with LtHb plus nitrite plus NO but not for the sample with only added NO. From the initial concentration of the standard GSNO, and allowing for the dilution within the tubing, the concentration of GSNO in the eluent from the LtHb sample can be directly calculated from the spectrum using the extinction coefficient and an integration of the GSNO elution band. Based on the absorbance and integrated intensity, we found that the amount of GSNO formed from the initially added GSH corresponds to less than 10% of the total number of hemes in the LtHb sample being active with respect to converting GSH to GSNO. The fact that (i) the LtHb sample is not fully converted to the NA intermediate under the conditions of this experiment, (ii) the NA intermediate for LtHb appears less stable than for HbA, and (iii) the time lag in adding the GSH after formation of the moderately unstable LtHb NA intermediate all indicate that the derived number underestimates the efficacy with which the intermediate generates GSNO from GSH. The low yield for the nitrite plus NO and the zero yield for the NO without nitrite also indicate that there were no issues of an oxygen leak in the presence of residual NO because under those conditions one would observe a much larger yield of GSNO. GSNO formation from HbA under similar conditions was reported previously (14).

## DISCUSSION

The tendency of the iron centers in Hb to gain or lose electrons is reflected by their intrinsic redox potentials. These Hb redox potentials are physiologically important because iron oxidation states influence both axial ligand binding and redox-mediated reactivity. The current study confirms the previously reported findings (27, 57) that the redox potential of LtHb is very high relative to both HbA and myoglobin. The present study also shows that the isolated large and small subunits have very different redox potentials. The redox potentials of the individual subunits are both more positive than the redox potentials of HbA and myoglobin. The redox potential associated with the larger LtHb subunits is dramatically higher than all of the others. The large difference in the redox of the isolated subunits is consistent with the several manifestations of functional heterogeneity seen in the intact polymer. Future studies will examine the reactivity of the individual subunits to determine whether the differences in redox potential match with differences in redox-sensitive reactions such as the nitrite reductase, reductive nitrosylation, and reduction by l-Cys.

The high redox potential of LtHb is a plausible explanation for several functional observations. The rates of reduction by l-Cys of the met derivative of LtHb and HbA with respect to l-Cys are consistent with the higher redox potential for LtHb in that the rate of reduction for the LtHb is considerably faster. The low fraction of ferric hemes in LtHb that are reduced by the l-Cys could be a reflection of heterogeneity in either the redox potential or accessibility of l-Cys for the different heme sites. Similarly the report of reduced rates for the nitric oxide dioxygenase reaction in both LtHb (17, 18) and another large polymeric worm hemoglobin (26) is also consistent with either a very high redox potential or low accessibility of reagents into the distal heme pocket of these proteins for at least some sites, possibly the result of a reduced volume within the distal pocket relative to HbA and myoglobins.

Several studies have provided evidence that the rate of the nitrite reductase reaction of heme proteins scales inversely with redox potential (33, 34). That relationship accounts at least in part for the higher NR rate for the R state of HbA *versus* the T state (8, 10, 30–32, 34, 36, 62). Overall the present study supports the extension of these earlier findings to LtHb in that the overall rate of the NR reaction is much slower than for HbA, consistent with the ordering of the redox potentials. The rates for the autoreduction of the Fe^3+^NO derivatives (RN reaction) follow a similar pattern in that the LtHb derivative undergoes RN at a much faster rate than HbA as anticipated based on the ordering of the redox potentials. The clear cut biphasic nature of the NR time course for LtHb is again consistent with functional heterogeneity among the heme sites in LtHb with respect to redox potential as anticipated from the redox potentials from the isolated subunits. This functional heterogeneity for LtHb could allow it to function both as a highly stable oxygen transport protein and as an NO/nitrite-detoxifying agent (28, 63). The need for critically important dual functionalities in a single hemoglobin is a possible driving force for the evolution of subunits that require different redox properties.

In the presence of an excess of nitrite, the addition of stoichiometric amounts (with respect to heme) of NO to ferric LtHb results in the formation a spectroscopically distinct species that is very similar to the HbA species assigned to the NA intermediate that has previously been attributed to N_2_O_3_ bound to a ferrous heme (14, 41, 42). The present study provides more support for this assignment. The results obtained using DAF-FM under anaerobic conditions show that the addition of both nitrite and NO to metHb produces a population of Hb that is more effective at generation of the triazole derivative of the DAF-FM relative to the same samples without the nitrite. Both the intensity changes and the wavelength changes support that assessment. The small changes observed to the nitrite-free sample could be due to low levels of oxygen still being present or binding of some reagent to the DAF causing the small changes. These results are paralleled by the HPLC finding for HbA reported by us previously (14) and the present result for LtHb showing formation of GSNO when both NO and nitrite are present with metHb but not when just NO is present. That observation is consistent with another study showing that the addition of nitrite to a mixture of NO and metHbA enhances GSNO production (39). The EPR results show that although the absorption spectrum of the proposed NA intermediate for both proteins closely resembles that of the corresponding ferrous NOHb derivatives the derivative attributed to the NA intermediate does not appear to have a detectable EPR signature in the region of the ferrous NOHb derivatives of either HbA or LtHb. The reactivity of the NA intermediate under low oxygen conditions and the absence of an EPR signal indicative of a ferrous NOHb derivative along with the conditions under which the NA intermediate is generated are all consistent with assigning the NA intermediate species to a derivative with a heme-bound N_2_O_3_. The absorption spectrum is most consistent with the NA intermediate being a ferrous derivative.

Although both HbA and LtHb form the NA intermediate, there are clear differences in the efficacy with which this reaction can compete with RN. In the absence of the nitrite, a rapid RN reaction is observed for the LtHb. It is only when an excess of nitrite is added that there is an appreciable formation of the intermediate for LtHb for the pathway involving the addition of both nitrite and NO to the met derivative. At low nitrite levels, the RN reaction still dominates. That behavior is different from that observed for HbA (14) where even at low nitrite and NO concentrations the NA reaction dominates over RN (formation of the “intermediate” is used here as indicative of the NA reaction despite the NA reaction being described as having the N_2_O_3_ released from the heme). The HbA study supported a mechanism where the intermediate could form from either NO or nitrite binding first; however, in situations where there is not a large excess of nitrite, the NO binding first mechanism is claimed to be much more probable based on predictions from simulations (38). The very low affinity of nitrite for ferric heme in Hbs implies that nitrite-based reactivity might be comparably low. The present and earlier studies (14, 34, 42) indicate that nitrite reactivity toward ferric hemes can be quite high when NO is present. The likely mechanism (64, 65) is NO binding first followed by the reaction of nitrite with ferric NO/ferrous NO^+^ resonance structure. Results obtained in trehalose-derived glassy matrices (41) suggest that in the presence of an excess of nitrite where there is a substantial population of heme-bound nitrite it is likely that NO rapidly displaces the bound nitrite followed by the displaced nitrite within the distal heme pocket rapidly reacting with the newly formed ferric NO/ferrous NO^+^ to form the ferrous N_2_O_3_ derivative.

The reactivity difference between the two proteins can be attributable largely to the higher redox potential of LtHb, although the difference in nitrite accessibility to the heme site may also be a factor. The higher redox potential supports a faster RN reaction. As a consequence, under similar low nitrite conditions, formation of the intermediate from Fe^3+^NO is less likely for LtHb relative to HbA because of the relative rapidity with which the RN occurs for LtHb. The redox potential is not likely to be the complete story with respect to NA *versus* RN in that myoglobin, which has a lower redox potential than HbA, does not form the intermediate.^3^ The fact that the nitrite derivative of metMb is high spin compared with the low spin forms seen for HbA and LtHb suggests that factors such as the internal structure of the distal heme pocket impacting both volume and charge distributions may limit the formation of the NA intermediate.

## CONCLUSIONS

LtHb undergoes the same nitrite-based reactions as HbA but at a much reduced efficiency and with clear evidence of functional heterogeneity. As with metHbA, metLtHb in the presence of both nitrite and NO can result in the formation of a relatively stable spectroscopically distinct species that has been attributed to N_2_O_3_ bound to a ferrous heme. This species is referred to as the nitrite anhydrase intermediate. The lack of an observable EPR spectrum for this species in combination with its reactivity under low oxygen conditions support an assignment for a ferrous heme-bound N_2_O_3_. Unlike the situation for HbA, the rate of formation of this proposed NA intermediate for LtHb is slower than the rate of reductive nitrosylation. The reduced reactivity of LtHb both with respect to the nitrite reductase and nitrite anhydrase reactions relative to HbA suggests that under physiological conditions where NO levels are depleted as in endothelial dysfunction or in the event of NO scavenging LtHb as a possible HBOC may not be as effective as some PEGylated Hbs with respect to compensatory mechanism (35). The *in vivo* data to date indicate that LtHb can be effective with respect to oxygen delivery under conditions where NO is not depleted. In the event that the reduced NO/N_2_O_3_-generating capability of LtHb becomes a limiting factor for its use as an HBOC, supplementation with NO-releasing nanoparticles is a straightforward therapeutic solution. Sustained release of NO through biocompatible NO-releasing nanoparticles (66, 67) has been shown to offset vasoconstriction due to NO scavenging by HBOCs (68) and inhibit the inflammatory cascade triggered by rapid blood loss (69).

## Footnotes

3 C. J. Roche, M. S. Navati, and J. M. Friedman, unpublished results.

## Footnotes

2 The abbreviations used are:

HBOC: hemoglobin-based oxygen carrier
Bis-Tris: 2-[bis(2-hydroxyethyl)amino]-2-(hydroxymethyl)propane-1,3-diol
Lt: *L. terrestris*
HbA: human adult hemoglobin
NR: nitrite reductase
R: relaxed
T: tense
NA: nitrite anhydrase
GSH: glutathione
RN: reductive nitrosylation
GSNO: *S*-nitrosoglutathione
DAF-FM: 4-amino-5-methylamino-2′,7′-difluorescein
Mb: myoglobin.
